# An update: improvements in imaging perfluorocarbon-mounted plant leaves with implications for studies of plant pathology, physiology, development and cell biology

**DOI:** 10.3389/fpls.2014.00140

**Published:** 2014-04-23

**Authors:** George R. Littlejohn, Jessica C. Mansfield, Jacqueline T. Christmas, Eleanor Witterick, Mark D. Fricker, Murray R. Grant, Nicholas Smirnoff, Richard M. Everson, Julian Moger, John Love

**Affiliations:** ^1^Division of Plant and Microbial Sciences, School of Biosciences, University of ExeterExeter, UK; ^2^School of Physics, University of ExeterExeter, UK; ^3^Computer Science, University of ExeterExeter, UK; ^4^Department of Plant Sciences, University of OxfordOxford, UK

**Keywords:** perfluorocarbon, *Arabidopsis*, multi-photon, confocal, microscopy, imaging, perfluoroperhydrophenanthrene

## Abstract

Plant leaves are optically complex, which makes them difficult to image by light microscopy. Careful sample preparation is therefore required to enable researchers to maximize the information gained from advances in fluorescent protein labeling, cell dyes and innovations in microscope technologies and techniques. We have previously shown that mounting leaves in the non-toxic, non-fluorescent perfluorocarbon (PFC), perfluorodecalin (PFD) enhances the optical properties of the leaf with minimal impact on physiology. Here, we assess the use of the PFCs, PFD, and perfluoroperhydrophenanthrene (PP11) for *in vivo* plant leaf imaging using four advanced modes of microscopy: laser scanning confocal microscopy (LSCM), two-photon fluorescence microscopy, second harmonic generation microscopy, and stimulated Raman scattering (SRS) microscopy. For every mode of imaging tested, we observed an improved signal when leaves were mounted in PFD or in PP11, compared to mounting the samples in water. Using an image analysis technique based on autocorrelation to quantitatively assess LSCM image deterioration with depth, we show that PP11 outperformed PFD as a mounting medium by enabling the acquisition of clearer images deeper into the tissue. In addition, we show that SRS microscopy can be used to image PFCs directly in the mesophyll and thereby easily delimit the “negative space” within a leaf, which may have important implications for studies of leaf development. Direct comparison of on and off resonance SRS micrographs show that PFCs do not to form intracellular aggregates in live plants. We conclude that the application of PFCs as mounting media substantially increases advanced microscopy image quality of living mesophyll and leaf vascular bundle cells.

## INTRODUCTION

Advances in microscopy have made *in vivo* biological imaging increasingly important in recent years. A diverse palette of chemical labels and genetically encoded fluorescent reporters and biosensors, coupled with advanced microscopy techniques, including laser scanning confocal microscopy (LSCM), two-photon fluorescence (TPF) microscopy and label-free imaging techniques such as second harmonic generation (SHG) microscopy and stimulated Raman scattering (SRS) microscopy have enabled unprecedented analysis of living cell dynamics (Shaw and Ehrhardt, 2013). Despite these technical advances, tissues that are rich in airspaces, such as those of plant leaves or animal lungs, remain difficult to image because of the optical aberrations that result from the complex structure of such tissues. As these tissues are typically sites of active metabolism and often targets for pathogens, it is essential to develop relatively simple *in vivo* methods to circumvent these imaging problems.

**FIGURE 1:**
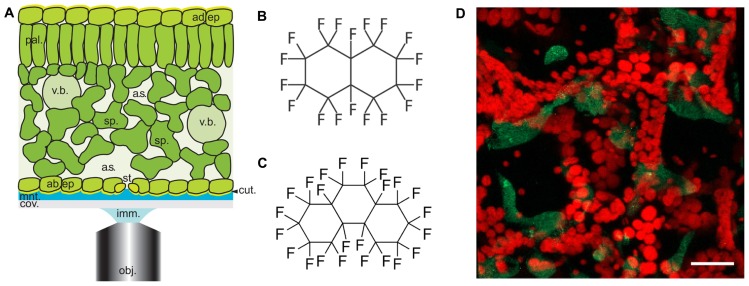
***Arabidopsis* leaf anatomy, chemical structures of perfluorocarbons used in this study and a typical image of *Pseudomonas* infected leaves. (A)** Diagrammatical representation showing the anatomical features of the *Arabidopsis* leaf in relation to the optical set-up. Abbreviations used are obj, objective lens; imm, immersion fluid; cov, coverslip; mnt, mountant; cut, cuticle; ad. ep, adaxial epidermis; st, stomatal pore; sp, spongy mesophyll; a.s, airspace; pal, palisade mesophyll; v.b, vascular bundle; ad. ep, adaxial epidermis. Cell walls are indicated by black lines (reproduced with permission from Littlejohn and Love, 2012). **(B,C)** chemical structures of **(A)** PFD and **(B)** PP11. **(D)** Z-stack reconstruction of GFP-expressing *Pseudomonas syringae* pv. tomato DC3000 infected *Arabidopsis* leaf. GFP signal is shown in green and chlorophyll autofluorescence in red. Scale bar is 25 μm.

The spongy mesophyll of higher plant leaves is located adjacent to the lower epidermis, contains numerous airspaces and may be several cell layers thick (**Figure 1A**). These characteristics result in light refraction within the mesophyll and a progressive attenuation of light transmission through the tissue, producing optical aberrations that impair confocal image quality (Feijó and Moreno, 2004; Inoue, 2006; Cheng, 2006). When examining fixed leaves these aberrations are minimized because the fixatives infiltrate the tissue and minimize the optical phase transitions within. Previously, we have shown that it is possible to infiltrate living leaves with perfluorodecalin (PFD, **Figure 1B**) and thereby significantly improve the resolution of LCSM images of the mesophyll while affecting only minimally cellular physiology (Littlejohn et al., 2010). We have had numerous positive reports from users of PFD and some studies have now been published (Johnson et al., 2011; Knapp et al., 2012; Tschiersch et al., 2012; Carrión et al., 2013; Gest et al., 2013; Hoepflinger et al., 2013; Hutt et al., 2013; Mansfield et al., 2013; Wright et al., 2013).

In this paper, we report that the optical qualities of plant mesophyll can be further improved by infiltration with perfluoroperhydrophenanthrene (PP11, **Figure 1C**), a perfluorocarbon (PFC) that has a refractive index that is better matched to that of living cells. To measure the improvement in image quality more objectively, we developed a method of autocorrelation that quantifies the sharpness of the images acquired at varying depths within the leaf. Finally, using SRS microscopy we have shown that both PFD and PP11 are undetectable in living cells, but infiltrate the mesophyll airspaces homogenously. Furthermore, SRS imaging of PFCs *in vivo* may be used to delimit the “negative space” within plant leaves, i.e., the area within the leaf that is involved in gaseous exchange and pathogen invasion. We have advocated the application of PFC mounting of samples to studies of pathogenesis in plants and experiments to date are promising. For example, **Figure 1D** shows an example image of an *Arabidopsis thaliana* leaf infected with the pathogenic bacterium, *Pseudomonas syringae* pv. tomato (DC3000 expressing GFP), taken under the same conditions as those used in Hutt et al. (2013).

## MATERIALS AND METHODS

### PLANT CULTURE AND SAMPLE MOUNTING

*Arabidopsis thaliana* (Col-0 ecotype) and transformants that stably and constitutively express a cytoplasmically localized “Venus” yellow fluorescent protein (SEYFP-F46L; Nagai et al., 2002) were used in this study. Seeds were surface sterilized for 3 min with 70% ethanol and then for 5 min with 10% sodium hypochlorite. Seeds were washed five times in water and suspended in 0.1% agar. Seeds were stratified at 4°C, in the dark, for 48 h before being sown on compost and grown at 20°C, in a 16 h/8 h light/dark photoperiod.

Mature leaves were excised from plants aged approximately 3-weeks and sections floated in H_2_O, PFD, or PP11 for 5 min according to the methods described in Littlejohn et al. (2010), Littlejohn and Love (2012). Samples were mounted in the same medium and imaged by LSCM, TPF, SHG, and SRS microscopy.

### LSCM IMAGING

**FIGURE 2:**
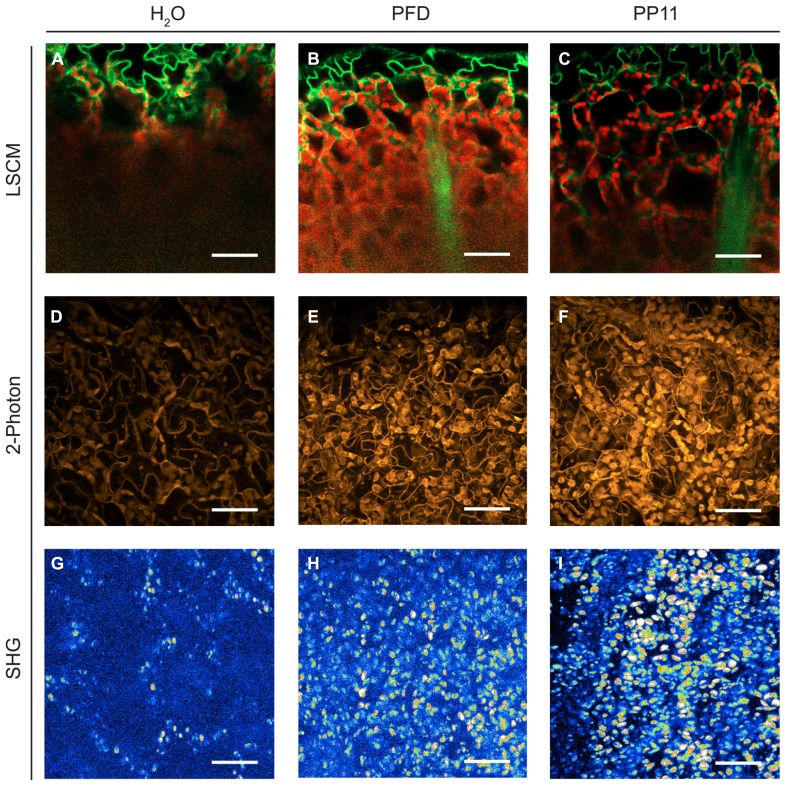
**Perfluorocarbon mounting media used with three modes of microscopy. (A–C)** LSCM images captured from 1 μm resolution Z-stacks taken of samples mounted in H_2_O, PFD and PP11 respectively. Images were generated by using the “cut” function in Zeiss LSM Image Browser software, where the plane presented represents a cut through the entire z-stack from top to bottom taken at an angle of -24°. The images therefore show a progression through the stack from top to bottom. Signal was collected from 518 to 604 nm for Venus, and is displayed in green and at 647–690 nm for chlorophyll, shown in red. **(D–F)** 2-photon micrographs taken of samples mounted in H_2_O, PFD, and PP11 respectively. **(G–I)** SHG micrographs taken of samples mounted in H_2_O, PFD, and PP11 respectively. **(D–I)** are presented as projections of z-stacks. Scale bars are 50 μm.

**FIGURE 3:**
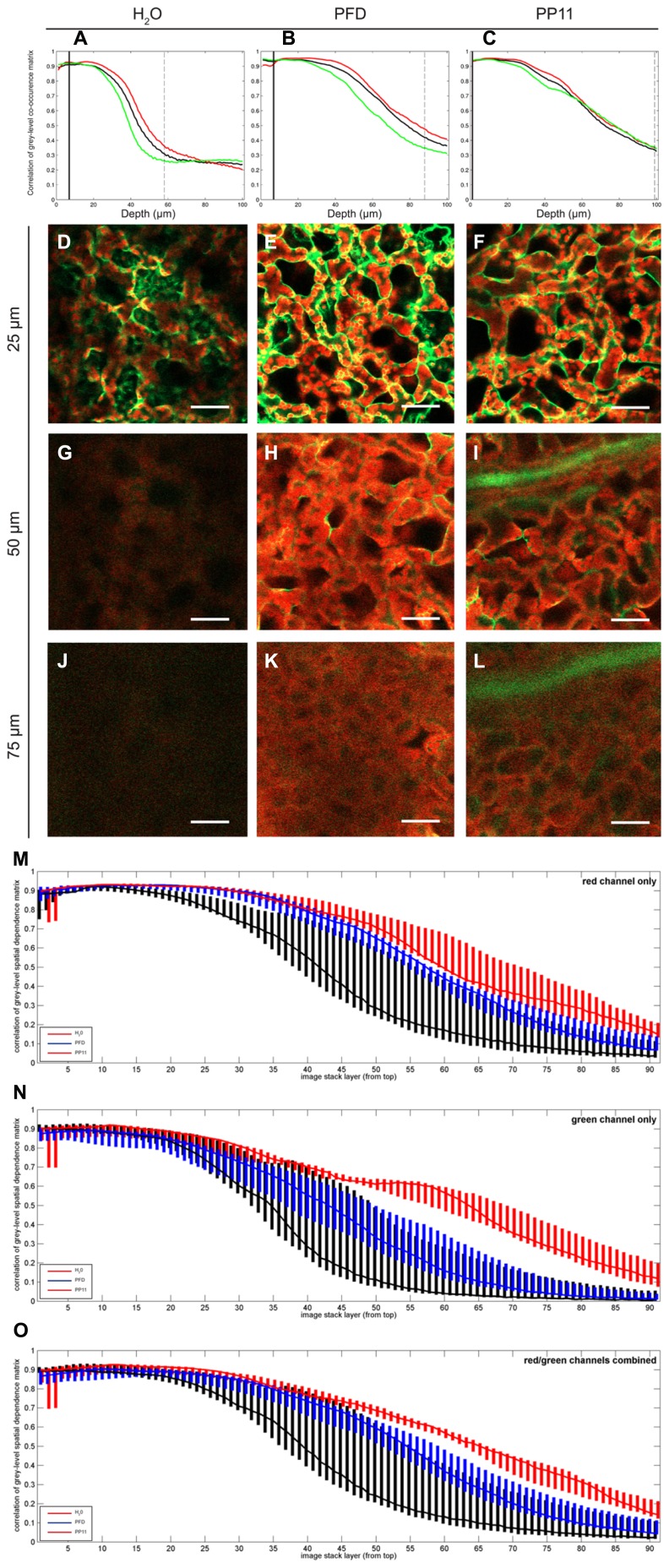
**Quantification of image deterioration with depth. (A–C)** typical autocorrelation results for single samples mounted in H_2_O, PFD, and PP11 respectively and imaged as z-stacks 100 μm deep. **(D–F)** representative images taken at a depth of 25 μm from samples mounted in H_2_O, PFD, and PP11 respectively **(G–I)** representative images taken at a depth of 50 μm from samples mounted in H_2_O, PFD, and PP11 respectively. Signal was collected from 518 to 604 nm for Venus, and is displayed in green and at 647–690 nm for chlorophyll, shown in red. **(J–L)** representative images taken at a depth of 75 μm from samples mounted in H_2_O, PFD, and PP11 respectively. **(M–O)** range and median plotted for all samples (*n*=5) mounted in H_2_O, PFD, or PP11 for red, green, and total signal respectively. Scale bars are 50 μm.

Confocal imaging was performed using a Zeiss Axiovert 510 Meta LSCM equipped with a 40x/1.30 oil DIC immersion C-Apochromat lens. Immersion medium was Zeiss immersol. Light paths and wavelengths were controlled by a 458/514 nm dichroic mirror. The pinhole was set at 70 μm. Images were integrated and processed using Zeiss 510 software. Images of Venus and chlorophyll fluorescence in intact *Arabidopsis* leaves were collected with excitation at 514 nm using a 30 mW argon laser, 6.1 A, 21.8% transmission intensity. Emission was recorded at 518–604 nm for Venus and at 647–690 nm for chlorophyll. Z-stacks containing 100 z-planes taken with 1 μm step size were collected for each of five samples incubated in PFD, PP11, or H_2_O for 5 min before imaging and mounted in the same medium for imaging. Figures were assembled in Adobe Indesign. **Figure 2** LSCM images were generated by using the “cut” function in Zeiss LSM Image Browser software, where the plane presented represents a cut through the entire z-stack from top to bottom taken at an angle of -24°. The images therefore show a progression through the stack from top to bottom. Single z-planes are presented in **Figure 3**. GFP-expressing *Pseudomonas* (made according to Lambertsen et al., 2004) and chlorophyll shown in **Figure 1D** were excited respectively with 488 and 633 nm laser lines and emission captured from 505 to 570 nm (GFP) and 647–711 nm (chlorophyll). The GFP-*Pseudomonas* Z-stack projected in **Figure 1D** was 30 μm deep, with a 0.25 μm step size and was captured with a C-Apochromat 63x/1.2W Corr M27 lens. The projection was made in Zeiss LSM510 software.

### RAMAN SPECTROSCOPY

Prior to SRS imaging the Raman spectra of the PFCs were obtained using a Renishaw RM100 Raman microscope (Renishaw plc, UK), with a 785 nm diode laser and 1200 line/mm spectral grating, giving a spectral resolution of 1 cm^-1^.

### STIMULATED RAMAN SCATTERING MICROSCOPY

Stimulated Raman scattering microscopy required two, pulsed laser beams; one at a longer wavelength (Stokes beam) and the other at a shorter wavelength (pump beam). The difference between the wavelengths of the pulsed lasers was tuned to correspond to the energy of a Raman vibration of interest. In our system a 1064 nm pico-second laser (PicoTrain HighQ laser) provided the Stokes beam and the output from an optical parametrical oscillator OPO (Levante Emerald APE) – pumped by the frequency doubled output of the picoTrain laser – provided the pump beam. The pump beam was tuned to a wavelength of 991.4 nm, which excited the C–F vibrations at 687.5 cm^-1^.

The amplitude of the Stokes beam was modulated at 1.7 MHz using an EOM. When SRS occurred within the sample, a modulated decrease in pump beam intensity [stimulated Raman loss; Moger et al. (2012)] was detected.

A modified laser scanning confocal microscope (Fluoview 300 IX71 Olympus) was used for imaging the SRS. The objective was a 60x 1.2 NA water immersion objective (UPlanS Apo Olympus). The transmitted light from the sample was collected with a 60x 1.0 NA water-dipping condenser and detected using a photo-diode with a 70 V reverse bias (FDS1010 thorlabs). The 1064 nm Stokes beam was blocked from reaching the photo-diode using a band pass filter coherent anti-Raman scattering (CARS; 890/220 nm, Chroma technologies). A lockin amplifier was used to separate out the modulated SRL signal and the output from this was connected to the computer and imaging software. Raman Image stacks were acquired at a 512 × 512 pixel resolution and a 256 μm × 256 μm scan area with 1 μm separation between optical slices.

### TWO-PHOTON FLUORESCENCE AND SECOND HARMONIC GENERATION MICROSCOPY

Two-photon fluorescence and SHG were performed using the customized microscope described above. Excitation was provided by mode-locked femtosecond Ti:sapphire oscillator (Mira 900D; Coherent, USA) which produced 100-fs pulses at 76 MHz. The central wavelength of the fs beam was 800 nm with an average power at the sample of 5–30 mW. TPF and SHG were spectrally separated from the 800 nm excitation beam by a dichroic mirror (670dcxr; Chroma Technologies). After this, different bandpass filters were used to enable either TPF signal (CG-BG-39-1.00-1 and F70-500-3-PFU; CVI Melles Griot, UK) or SHG signal (F10-400-5-QBL; CVI Melles Griot, UK) to reach the Hamamatsu R3896 photomultiplier tube.

### QUANTIFICATION OF IMAGE CLARITY IN THE Z-AXIS

The clarity of each image in relation to its position in the z-axis (i.e., into the tissue) was quantified using textural analysis. For each image, the “green” and “red” color channels were analyzed separately and in combination. A gray-scale spatial dependence matrix (Haralick et al., 1973), labeled “M,” was constructed from the intensities (ranging from 0 to 255) of each pixel that composed each image and for each channel. The element M**_r,c_, at row *r* and column *c* of this matrix is a count of how many times a pixel with intensity *r* has a pixel of intensity* c* in its immediate neighborhood, defined as the 8 pixels surrounding the measured pixel. Normalizing this matrix resulted in a set of joint probabilities of pixels with intensities *r* and *c* within the neighborhood. The correlation value associated with this normalized matrix (see Haralick et al., 1973) gives a measure of how closely correlated the intensity of a given pixel is with those of its neighboring pixels. The correlation measure was calculated for each image in acquired z-stacks, 100 μm deep, with a 1 μm z-resolution for leaves mounted in H_2_O, PP11 and PFD stacks, and separately for the red, green, and combined red/green channels.

## RESULTS

### PERFLUOROCARBON MOUNTING MEDIA IMPROVE THE OPTICAL RESOLUTION OF DIFFERENT MODES OF LASER SCANNING MICROSCOPY

We compared the image resolution of micrographic z-stacks acquired using LSCM, TPF, and SHG microscopy (**Figure 2**). TPF and SHG are non-linear optical techniques, which involve the simultaneous absorption of two or more photons. All these techniques are intrinsically confocal and generally use infra-red lasers instead of visible or UV lasers to excite fluorophores (Nandakumar et al., 2009). These techniques are considered advantageous compared to single-excitation confocal microscopy because they can deliver improved imaging depths within scattering tissues and reduced photodamage within samples.

Two-photon fluorescence is similar to single photon fluorescence, but it requires the simultaneous absorption of two near infra-red photons, rather than a single UV or visible spectrum photon, to generate a fluorescent signal (Diaspro and Sheppard, 2002).

Second harmonic generation involves the simultaneous absorption of two near infra-red photons and the emission of a single visible photon with half the wavelength of the infra-red photons. This process only occurs in structures which lack inversion symmetry. In plant tissues these structures include cellulose and starch grains (Mizutani et al., 2000; Brown et al., 2003; Cox et al., 2005).

Under all modes of microscopy tested, PP11 and PFD outperformed H2O as an *in vivo* mounting medium, by infiltrating the mesophyll airspaces and smoothing the optical transitions within the mounted leaves. Moreover, we noted that PP11 outperformed PFD, which we ascribe to the refractive index of PP11 being closer to that of living cells compared to PFD. We were routinely able to acquire images from a depth of 100–135 μm within the leaf by LSCM and two photon imaging using PP11 as a mounting medium, which is greater than half the thickness of a leaf of a 3 week-old *Arabidopsis* plant and tests in with rice leaves allow imaging through the entire thickness of the leaf. The use of PFCs as a mounting medium also allowed the acquisition of SHG signals from chloroplast starch, which was not possible for samples mounted in H2O. This may, in itself, represent an important technique for the study of starch in leaves and statoliths in root cells.

To quantify the apparent advantage of using PFC mounting media to image within the mesophyll, we performed autocorrelation analysis on LSCM micrographs acquired at varying depths in *Arabidopsis* leaves. In this case, the fluorescence emission for both cytoplasmically localized Venus (Nagai et al., 2002) and chlorophyll were recorded. The pixel-by-pixel autocorrelation enabled an objective quantification of image quality. Using this method, we noted a wavelength-dependent improvement in resolution when samples were mounted in PFCs, compared to H_2_O (**Figure 3**). Images recorded deeper in the sample are more greatly affected by noise, which tends to be uncorrelated, and hence the correlation measure is low compared with the crisper images recorded closer to the surface. Median values for the autocorrelation demonstrate that PP11 and PFD outperform H_2_O and PP11 performs better than PFD, with a greater benefit seen in imaging Venus, compared with chlorophyll (**Figures 3M–O**). Most interestingly, the use of PFCs as mounting medium reduced the range of autocorrelation values obtained, showing that the images acquired when samples are mounted in PFD or in PP11 are not only clearer, but more consistent between replicate samples.

**FIGURE 4:**
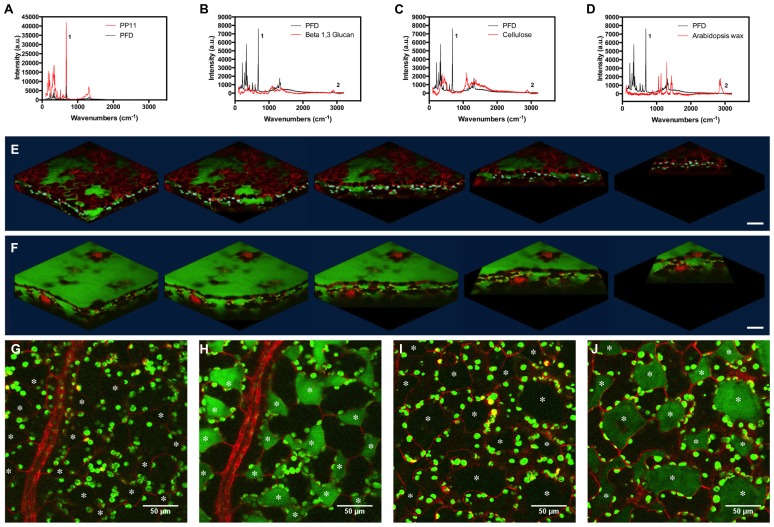
**Stimulated Raman scattering imaging of perfluorocarbons *in vivo*. (A–D)** Raman spectra taken at 785 nm, comparing spectra of PFD and **(A)** PP11, **(B)** Beta 1–3 glucan, **(C)** cellulose, and **(D)** hexane extracted *Arabidopsis* leaf wax. Peaks labeled one and two were used to tune imaging to C–F and C–H bonds respectively. **(E,F)** SRS images representing Z-stacks taken through *Arabidopsis* leaves mounted in **(E)** PFD and **(F)** PP11. **(G,H)** show respectively, off and on resonance images of an *Arabidopsis* leaf mounted in PP11. **(I,J)** show respectively, off and on resonance images of an *Arabidopsis* leaf mounted in PFD. Scale bars are 50 μm. Asterisks denote airspaces.

Normal cytoplasmic streaming and chloroplast movement was observed in all the experiments performed in this investigation, which is consistent with previous observations that the use of PFCs has a minimal effect on leaf physiology compared to mounting leaf samples in H_2_O (Littlejohn et al., 2010). This is also evidenced by the differences in chloroplast position seen between z-stacks represented in **Figures 4G–J**.

### STIMULATED RAMAN SCATTERING IMAGING OF PERFLUOROCARBON DISTRIBUTION

TPF and SHG and SRS are non-linear optical techniques, which involve the simultaneous absorption of two or more photons. All these techniques are intrinsically confocal and generally use infra-red lasers instead of visible or UV lasers to excite fluorophores.

Stimulated Raman scattering is a chemical-specific technique which relies on stimulating Raman active molecular bond vibrations. SRS requires stimulation by two laser beams at different wavelengths (pump and Stokes beams), with the difference in wavelength between the two beams set to correspond to the energy of the molecular bond vibration of interest. When this condition is met, SRS occurs and results in a loss of intensity in the higher energy pump beam (stimulated Raman loss) and an equal increase in intensity in the Stokes beam (stimulated Raman gain). This process is detected by modulating one of the beams and detecting the modulations in intensity in the second beam using a lockin amplifier (Freudiger et al., 2008).

Perfluorocarbons readily infiltrate leaf airspaces. To ascertain whether PFCs remain localized in the airspaces of the mesophyll or are capable of also penetrating living cells, we imaged the PFC distribution in infiltrated *Arabidopsis* leaves using SRS. Prior to imaging, we demonstrated that Raman spectra may be used to identify specific peaks, which allow the C–F bonds in the PFC and the C–H bonds found in *Arabidopsis* surface waxes, in cellulose and in β-1-3-glucan to be easily distinguished (**Figures 4A–D**). PFC mounting media are therefore compatible with Raman-based imaging of biological molecules (**Figures 4E,F**). Images of the “ON” and “OFF resonance confirmed that PFCs was homogeneously distributed in the airspaces between mesophyll cells (Littlejohn et al., 2010). However, the sensitivity of this technique enables us also to confirm that PFCs do not appear to form intracellular aggregates, and are therefore unlikely to penetrate beyond the apoplast into the protoplasm (**Figures 4G–J**). From a structural perspective, SRS imaging of PFC distribution in leaves has the added advantage of delimiting the mesophyll airspaces, potentially enabling a more intricate understanding and modeling of gas flow dynamics in leaves. The chloroplasts appear in both the on and off resonance SRS images due to their strong two-photon absorption (TPA). In this process they are absorbing one photon originating from the pump beam (either 991 or 989 nm on or off resonance) and one photon from the stokes beam (1064 nm). As the absorption spectrum of the chloroplasts is so broad, tuning the wavelength of the excitation laser does not affect the strength of the chloroplast signal.

Some of the chloroplasts may appear to be on the extracellular side of the red lines (cell walls) delineating the cells in the images.

This is due to the strong out-of-focus signal from chloroplasts; normally 2-photon excitation is constricted to a small, defined volume and therefore no pinhole is used to filter out-of-focus information. However, as explained, the absorption of the chloroplasts is so strong that where the chloroplast signal overlaps with that of the PFC, this is most likely an imaging artifact due to the strength of the TPA signal from the chloroplasts compared to the red fluorescence in the cells and the SRS from PFC, particularly as the phenomenon is seen in both on and off images. As the Chloroplast signal is very intense signal from adjacent image frames could be leaked into selected image frame (resolution in z direction is slightly worse than 1 um which is the image step size here). This has the effect of making the chloroplasts appear to be the “wrong” side of the red cell walls.

We cannot categorically exclude the possibility that PFC have penetrated the cells, but consider it to be unlikely as otherwise the TPS from the PFC would be much more ubiquitously distributed.

## DISCUSSION

In this investigation, we show that mounting living leaves in the PFCs, PFD, and perfluoroperhydrophenanthrene (PP11) quantifiably improves the clarity and consistency of images acquired from the mesophyll, for a number of laser-based microscopy techniques including LSCM, 2-photon microscopy, SHG, and SRS.

PFD and PP11 are non-toxic and possess a great carrying capacity for O_2_ and CO_2_. PFCs are not miscible with aqueous solutions, which are a disadvantage when trying to deliver bioactive compounds to cells in the leaf, however, this does suggest that PFCs may be of great use as they will not dilute metabolites or signaling molecules present in the cell wall, which could be easily perturbed by aqueous mounting media.

Both PFCs used in this investigation have low surface tensions (19.3 dyne cm^-1^ for PFD and 21.6 dyne cm^-1^ for PP11 compared to 72.8 dyne cm^-1^ for H_2_O; Sargent and Seffl, 1970) that are lower than the 25–30 dyne cm^-1^ required to passively overcome the stomatal barrier (Schönherr and Bukovac, 1972) and readily infiltrate the apoplastic space. This infiltration smooths the optical phase transitions within the mesophyll, resulting in reduced noise and quantifiably clearer images. Moreover, the properties that PFD has displayed for mesophyll – easy infiltration into the tissue, significant improvement in *z*-plane resolution and non-toxicity – may be exploited for more general, *in vivo* imaging of air-filled or heavily vascularised animal tissues, such as insect spiracles or vertebrate lung, where gaseous exchange is also important and which are primary target for microbial infection. Similarly, PFD may be used advantageously for the perfusion and imaging within organ cavities.

The improvement in image clarity obtained by mounting samples in PP11 is greater than for PFD, which we ascribe to a closer matching of the refractive index of PP11 (1.334) with that of leaf tissue, which has been estimated as 1.4–1.6 depending on wavelength of incident light (Paillotin et al., 1998; Feret et al., 2008) compared to that of PFD (1.313). This was shown by autocorrelation analysis of pixel intensities in LSCM images. This analysis also demonstrated that the use of PP11 is particularly advantageous for imaging shorter wavelengths (i.e., the “green” channel; **Figure 3**). Increased fluorescence transmission of shorter wavelengths may have important advantages when more than one fluorophore is imaged simultaneously, for example in analyses that require Forster resonance energy transfer (FRET) or co-localisation.

We anticipate that the general improvement in image quality conferred by mounting in PFC media could be further enhanced through an even closer match between the refractive indices of living cells and the PFC mounting medium. 2,2′-thiodiethanol (TDE) has previously been used in this fashion (Staudt et al., 2007); in aqueous solution at varying concentrations, TDE provides a suite of colorless mounting media with tuned refractive indices from 1.33 to 1.52. Although TDE is not compatible with living samples and used preferentially with fixed specimens, it raises the possibility of a tuneable mounting system composed of two PFCs, each with refractive indices that bracket those of living specimens. These complementary PFCs may then be combined in set proportions to make a mounting medium in which the refractive index is matched to that of any sample.

The presence of C–F bonds in PFCs can be readily distinguished from the C–H bonds found in biological molecules. Consequently, PFCs does not impair the imaging of biological molecules by label-free imaging techniques such as CARS microscopy and SRS microscopy (Mansfield et al., 2013). The C–F bond itself can be exploited to visualize the distribution of PFC in biological material. In this investigation, we have exploited this distinction to generate contrast images of the “negative space” within the mesophyll. Images of the apoplast throughout development will enable a better understanding of leaf expansion and growth. Moreover, is will be possible to use such techniques to develop refined models of the airspaces within leaves, and better understand the constraints of gaseous fluxes within.

## CONCLUSION

We have shown that mounting living leaves in PFD and perfluoroperhydrophenanthrene (PP11) improves image resolution for a number of laser-based microscopy techniques including LSCM, TPF, SHG, and SRS microscopy. These compounds allow greater *z-*axis penetration, resulting in clearer micrographs. In addition, PFCs may be used as label-free contrast agents to image the internal architecture of leaves, and enable a more precise understanding of the structural changes that occur during leaf development. As the mesophyll is a primary target for pathogenesis in plants, this technique may also be used to observe the processes of infection deep within the mesophyll. More generally, the use of PFCs as mounting media may be applied to other tissues rich in airspaces, such as animal lungs.

## AUTHOR CONTRIBUTIONS

George R. Littlejohn, Mark D. Fricker, Murray R. Grant, Nicholas Smirnoff, Julian Moger, Jacqueline T. Christmas, Richard M. Everson, and John Love designed the research; George R. Littlejohn, Eleanor Witterick, Jessica C. Mansfield, and Julian Moger performed the experiments; George R. Littlejohn, Eleanor Witterick, Jessica C. Mansfield, Jacqueline T. Christmas, Julian Moger, and John Love analyzed the data; George R. Littlejohn, Jessica C. Mansfield, Jacqueline T. Christmas and Jessica C. Mansfield and John Love wrote the manuscript.

## Conflict of Interest Statement

The authors declare that the research was conducted in the absence of any commercial or financial relationships that could be construed as a potential conflict of interest.
